# Loss of FBP1 promotes proliferation, migration, and invasion by regulating fatty acid metabolism in esophageal squamous cell carcinoma

**DOI:** 10.18632/aging.103916

**Published:** 2020-11-21

**Authors:** Yi He, Rong Hua, Bin Li, Haiyong Gu, Yifeng Sun, Zhigang Li

**Affiliations:** 1 Department of Thoracic Surgery, Shanghai Chest Hospital, Shanghai Jiao Tong University, Shanghai, China

**Keywords:** FBP1, ESCC, miR-18b-5p, fatty acid metabolism, proliferation

## Abstract

Esophageal squamous cell carcinoma (ESCC) is one of the most common cancers in China. Recent studies have shown fatty acid metabolism is involved in the progression of various cancers through regulating the function of various types of cells. However, the relationship between fatty acid metabolism and tumorigenesis of ESCC remains unclear. Here, in this study, the expression of FBP1 was dramatically decreased in ESCC tissues compared with the adjacent non-ESCC tissues. The cell proliferation, migration, invasion and fatty acid metabolism were evaluated in ESCC cells using transfection of shFBP1 vectors. We found loss of FBP1 promoted ESCC cell proliferation, migration and invasion, which correlated with the activated fatty acid metabolism *in vitro*. Moreover, the content of phospholipids, triglycerides, neutral lipids and the protein expression levels of fatty acid metabolism related FASN, ACC1 and SREBP1C proteins were significantly increased following down-regulation of FBP1. Furthermore, FBP1 was found to be directly targeted by miR-18b-5p in ESCC cells. In addition, miR-18b-5p inhibitor treatment obviously reversed the increased fatty acid metabolism induced by loss of FBP1 in ESCC cells. These findings explored a detailed molecular mechanism of tumorigenesis and progression of ESCC and might provide a potential novel method to treat ESCC in clinic.

## INTRODUCTION

Esophageal cancer (EC) is a malignant tumor that occurs in esophageal epithelial cells [1]. EC ranks sixth in global cancer mortality and the EC patients in China account for about 50% of the total number of cases worldwide [2]. There are two main types of EC: esophageal squamous cell carcinoma (ESCC) and esophageal adenocarcinoma (EA) [3]. The TNM stage of ESCC complied with the criteria of the TNM system developed by the American Joint Committee on Cancer (AJCC). The TNM staging system classifies ESCC by the size and extent of the primary tumor, involvement of regional lymph nodes, and the presence or absence of distant metastases. T describes the size of the primary tumor and whether it has invaded various nearby tissues, including lamina propria mucosa, muscularis mucosa, esophageal adventitia and adjacent tissues. N describes regional lymph nodes that are involved, and M describes distant metastasis. Due to the shortage of specific symptoms and effective early diagnosis, ESCC is often diagnosed in advanced stages [4, 5]. At present, surgery, chemotherapy and radiotherapy are the main treatments for ESCC [6, 7]. However, the five-year survival rate of the ESCC patients is only near 15%, and most patients are less than one year after diagnosis [8, 9]. On the other hand, the postoperative complications, surgical trauma, and high postoperative mortality still disturb the ESCC patients [10, 11]. Therefore, it is urgent to investigate the detailed pathogenesis of ESCC and develop the novel treatments and methods.

Obesity is demonstrated to increase the incidence of ESCC and EA, and previous studies have shown that high-fat diet induces obesity to promote the progression of tumors in EA mice [12, 13]. Moreover, adipose tissue around the tumor could increase risk of progression of EA [14]. Furthermore, obesity or adipose tissue affected tumorigenesis and progression, including paracrine methods, involved in the tumor microenvironment of ESCC [15, 16]. Excess energy is the main cause of obesity, which involves the cellular glucose and fatty acid metabolism. Recent studies have shown that fatty acid metabolism, which is closely related to cancer cell function, regulates the invasion and metastasis of a variety of cancer cells [17–19]. However, whether the fatty acid metabolism affects the tumorigenesis and progression of ESCC remains to solve.

Fructose-1, 6-bisphosphatase 1 (FBP1), an enzyme in gluconeogenesis, is mainly involved in the catalysis reaction of fructose-1,6-diphosphate to produce fructose-6-phosphate. Previous studies have found that FBP1 abnormal expression or loss of function is observed in various malignant tumors such as breast cancer [20], hepatocellular cancer [21], pancreatic cancer [22], and lung cancer [23]. In addition, low expression of FBP1 was closely related to the drug resistance and the postoperative recurrence of the cancer [24, 25]. Consistently, we also found the FBP1 expression was down-regulation in ESCC patients. However, the relationship between FBP1 and fatty acid metabolism in the tumorigenesis and progression of ESCC, and the potential detailed molecular mechanisms remain unknown. In this study, we found loss of FBP1 promoted ESCC cell proliferation, migration, and invasion *in vitro*, which was involved in the regulation of miR-18b-5p. Moreover, the interaction of miR-18b-5p and FBP1 could regulate cell function through inhibiting fatty acid metabolism in ESCC cells. These findings explored a detailed molecular mechanism of tumorigenesis and progression of ESCC and might provide a potential novel method to treat ESCC in clinic.

## RESULTS

### Expression of FBP1 in ESCC tumor tissues

**Figure 1:**
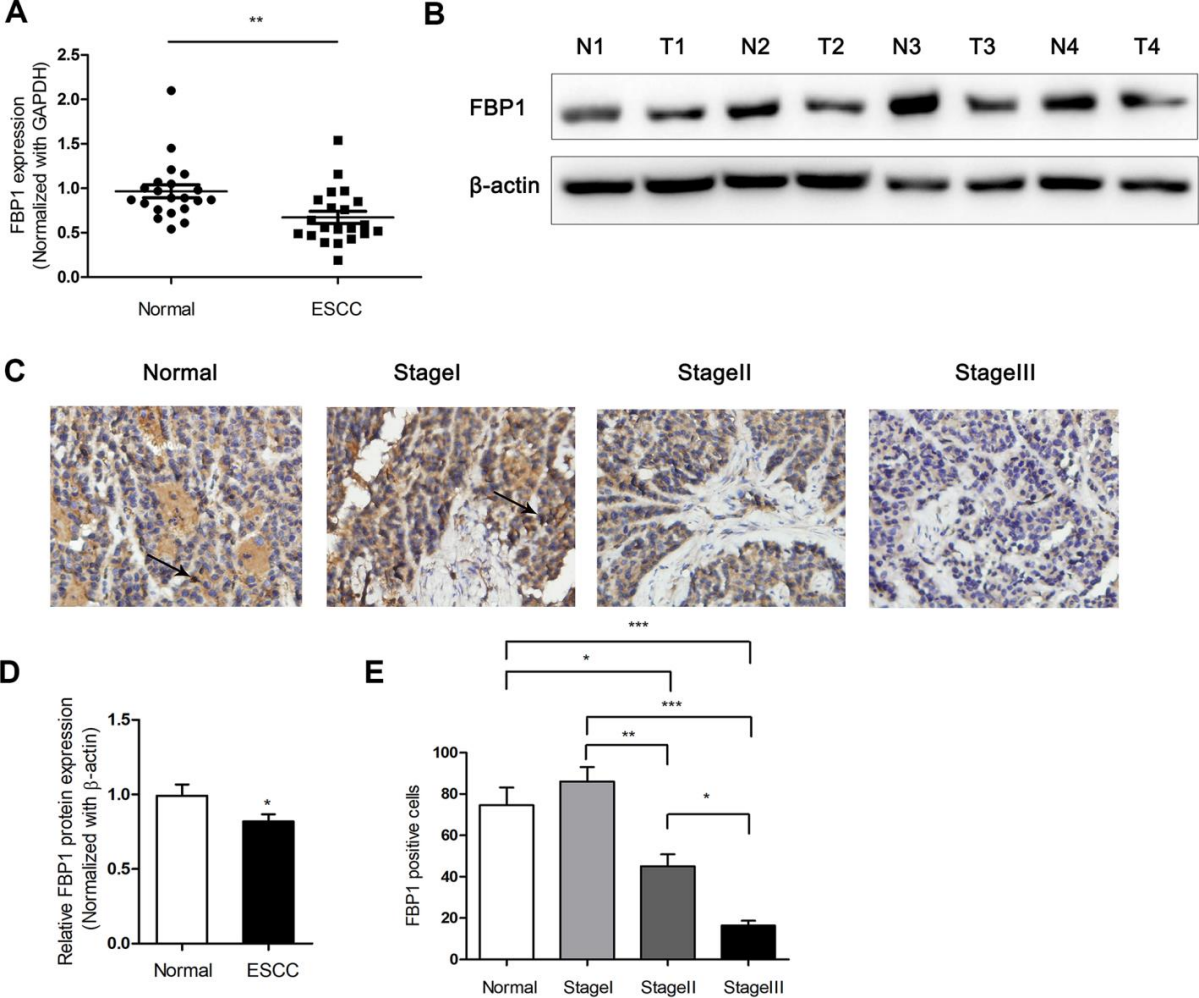
**Down-regulated expression of FBP1 was determined in ESCC tissues.** (**A**) Changes in FBP1 expression between ESCC tissues and adjacent non-ESCC tissues. (**B**, **D**) FBP1 protein expression and quantification were determined between ESCC tissues (T) and adjacent non-ESCC tissues (N). (**C**, **E**) Immunohistochemical analysis and quantification of FBP1 staining (200×) were performed in adjacent non-ESCC tissues and different TNM stage of ESCC according to the latest American Journal of Critical Care (AJCC) guide. *p< 0.05, **p< 0.01, ***p< 0.001.

To investigate the expression of FBP1 in ESCC tumor tissues, the FBP1 mRNA and protein levels were detected in ESCC tissues and adjacent non-ESCC tissues. As shown in Figure 1A, lower FBP1 mRNA levels were observed in 21 ESCC tissues compared with the FBP1 expression in 21 adjacent non-ESCC tissues (P<0.01). Next, we determined the protein expression of FBP1 between four ESCC tissues and the relevant adjacent non-ESCC tissues. The results demonstrated that FBP1 protein expression level was obviously decreased in ESCC tissues (Figure 1B, 1D). To further investigate the exact expression state of FBP1 in different TNM stage of ESCC, FBP1 expression was determined by immunohistochemistry. FBP1 expression was gradually decreased with the high stage of ESCC (Figure 1C, 1E). The expression levels of FBP1 in tissues of ESCC stageIII was barely observed, which suggested that low expression of FBP1 was correlated with the ESCC aggressiveness.

### Loss of FBP1 promoted ESCC cell proliferation, migration and invasion

**Figure 2:**
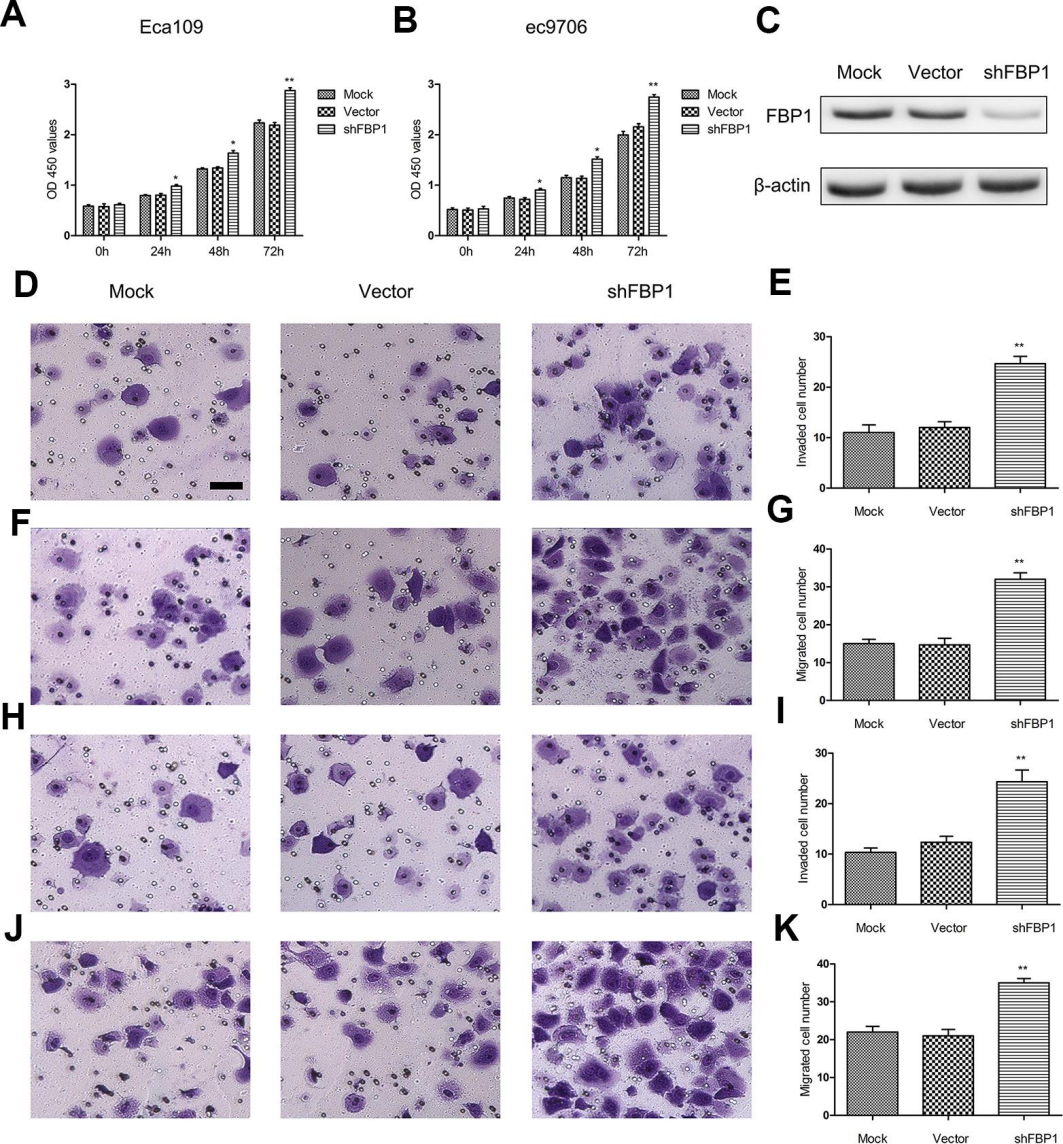
**Loss of FBP1 promoted ESCC cell proliferation, migration and invasion.** (**A**, **B**) The impact of FBP1 expression on ESCC cell proliferation as assessed using CCK-8 proliferation. (**C**) Validation of knockdown of FBP1 in Eca109 cells using shFBP1 transfection. (**D**, **E**) The impact of FBP1 expression on ESCC cell invasion was performed using shFBP1 transfection in Eca109 cells. (**F**, **G**) The impact of FBP1 expression on ESCC cell migration was performed using shFBP1 transfection in Eca109 cells. (**H**, **I**) The impact of FBP1 expression on ESCC cell invasion was determined using shFBP1 transfection in ec9706 cells. (**J**, **K**) The impact of FBP1 expression on ESCC cell migration was determined using shFBP1 transfection in ec9706 cells. scale bar= 20μm. Mock, the blank control group. Vector, the blank vector group. shFBP1, FBP1 shRNA vector group. *p< 0.05, **p< 0.01 VS Mock.

FBP1 was previously found to affect cell proliferation, migration and invasion in various cancer cells [26, 27]. To investigate the effect of FBP1 expression on ESCC cells function, Eca109 and ec9706 cells were treated with shFBP1 for 0, 24, 48, 72 h. The results showed that Loss of FBP1 significantly enhanced ESCC cell proliferation compared with the control (Figure 2A and 1B). We also showed the validation of knockdown of FBP1 in Eca109 cells following shFBP1 transfection for 48 h (Figure 2C). Moreover, to explore the effect of FBP1 expression on ESCC cell migration and invasion, Eca109 and ec9706 cells were treated with shFBP1 for 48 h. The cell migration (Figure 2D, 2E, 2H, 2I) and invasion (Figure 2F, 2G, 2J, 2K) were significantly increased by the treatment of shFBP1 compared with the control.

### Loss of FBP1 enhanced fatty acid metabolism in ESCC cells

**Figure 3:**
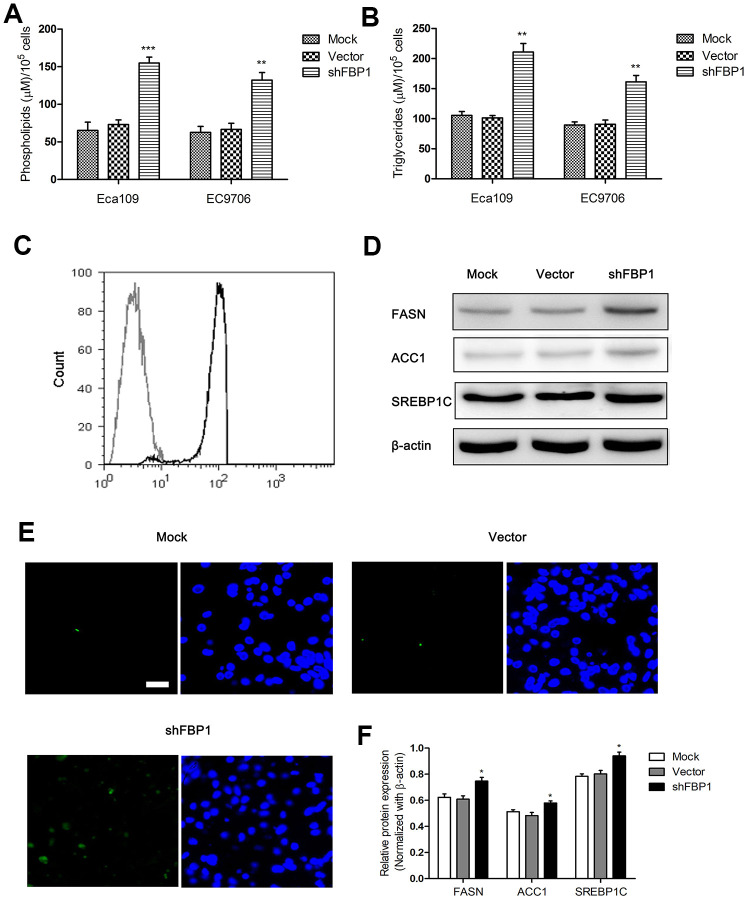
**Loss of FBP1 regulated fatty acid metabolism in ESCC cells.** (**A**) The impact of FBP1 expression on the content of phospholipids in ESCC cells. (**B**) The impact of FBP1 expression on the content of triglycerides in ESCC cells. (**C**) The neutral lipids content was detected by flow cytometry in Eca109 cells, gray, control; black, shFBP1. (**D**, **F**) The impact of FBP1 expression on the expression of FASN, ACC1 and SREBP1C was determined in ESCC cells. (**E**) The neutral lipids content was detected by staining with BODIPY 493/503 dye (green) and DAPI (blue) in Eca109 cells, scale bar= 20μm. Mock, the blank control group. Vector, the blank vector group. shFBP1, FBP1 shRNA vector group. **p< 0.01, ***p< 0.001 VS Mock.

Fatty acid metabolism was involved in cancer cell invasion and metastasis [28, 29]. To explore the relationship between FBP1 expression and fatty acid metabolism in ESCC cells, the cells were treated with shFBP1 for 48 h. The results showed that the content of phospholipids in ESCC cells was significantly increased following treatment with shFBP1 (Figure 3A). Furthermore, loss of FBP1 lead to an increase of the content of triglycerides in ESCC cells when compared with the control (Figure 3B). To further explore the alteration of fatty acid metabolism in ESCC cells, we determined the neutral lipids content using staining with BODIPY 493/503 dye. The results demonstrated that loss of FBP1 obviously enhanced the content of neutral lipids detected by flow cytometry (Figure 3C). On the other hand, the expression levels of fatty acid metabolism related FASN, ACC1 and SREBP1C protein were obviously increased following down-regulation of FBP1 compared with the control (Figure 3D, 3F). In addition, following staining with BODIPY 493/503 dye and DAPI, we found loss of FBP1 significantly increased the immunoreaction of BODIPY 493/503 dye compared with the control, which suggested that loss of FBP1 could enhanced the content of neutral lipids (Figure 3E).

### FBP1 was interacted with miR-18b-5p in Eca109 cells

**Figure 4:**
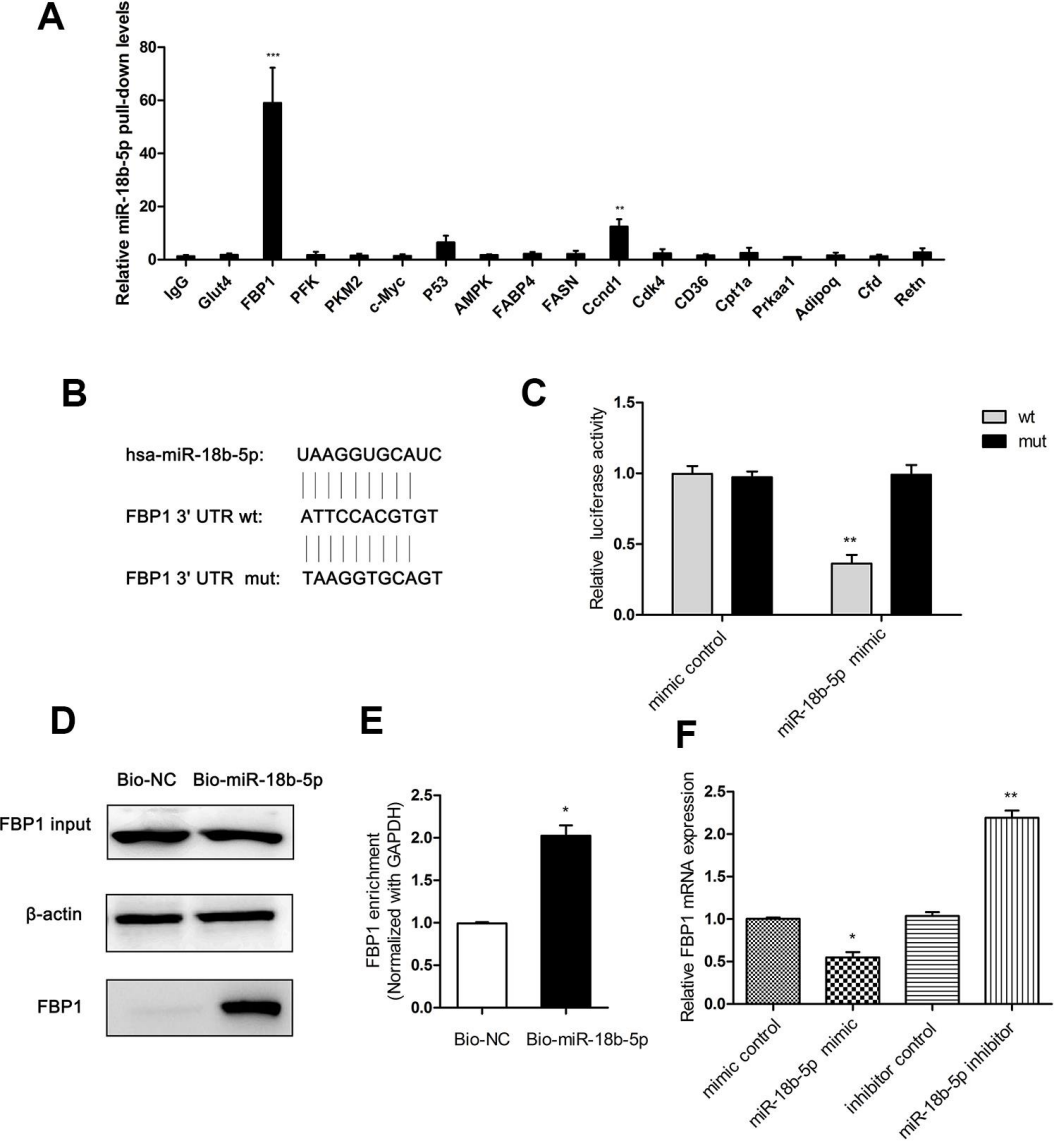
**FBP1 was interacted with miR-18b-5p in Eca109 cells.** (**A**) Cell lysates prepared from Eca109 cells were incubated with antibodies against IgG, Glut4, FBP1, PFK, PKM2, c-Myc, P53, AMPK, FABP4, FASN, Ccnd1, Cdk4, CD36, Cpt1a, Prkaa1, Adipoq, Cfd and Retn. The precipitated products were subjected to real-time PCR with primers amplifying miR-18b mRNA. Error bars, SD (n = 4). **p < 0.01, ***p < 0.001. (**B**) The prediction of miR-18b-5p and 3’UTR of FBP1 targeting site. (**C**) miR-18b-5p mimic directly targeted the wt of FBP1 in Eca109 cells. (**D**) The pulled down FBP1 in the Bio-miR-18b-5p transfected cells. (**E**) Enrichment of FBP1 transcripts in Bio-miR-18b-5p pull-down mRNA in Eca109 cells. (**F**) The FBP1 expression was determined following treatment with miR-18b-5p mimic or inhibitor. Wt, wild-type reporter plasmid containing FBP1 3’UTR; mut, mutations reporter plasmid in the same regions. Mimic control, the miR-18b-5p mimic negative control group. Bio-NC, biotin-labeled negative control. Bio-miR-18b-5p, biotin-labeled miR-18b-5p. Inhibitor control, the miR-18b-5p inhibitor negative control group. *p< 0.05, **p< 0.01 VS respective control.

MiR-18b-5p was previously demonstrated to promote cell invasion and metastasis in MCF-7/PR cells [30]. To investigate the effect of miR-18b-5p on the progression of ESCC, cell lysates from Eca109 cells were acquired and incubated antibodies against glucose and lipid metabolism-related proteins. Antibody against IgG was used as control. We found that the anti-FBP1 antibody precipitated significantly higher levels of miR-18b-5p (Figure 4A). To explore the detailed mechanism of miR-18b-5p regulating fatty acid metabolism in ESCC cells, the tentative interaction was predicted by the target prediction program Targetscan. The result showed that FBP1 was predicted to interact with miR-18b-5p from the target analysis (Figure 4B). To further testify the relationship between miR-18b-5p and FBP1 in ESCC cells, Eca109 cells were co-transfected with reporter plasmid contained wild type (wt) or mutant type (mut) of 3’UTR of FBP1 and miR-18b-5p mimic. The dual luciferase reporter assay showed that miR-18b-5p directly targeted the wt of FBP1 in Eca109 cells (Figure 4C). Moreover, to further explore the relationship between miR-18b-5p and FBP1 in ESCC cells, RNA pull-down analysis was performed in Eca109 cells. The results revealed that the Bio-miR-18b-5p pulled down FBP1 in Eca109 cells (Figure 4D and 4E). Moreover, the relative expression of FBP1 was decreased after treatment with miR-18b-5p mimic, and miR-18b-5p inhibitor significantly increased FBP1 relative expression compared with the respectively control (Figure 4F).

### miR-18b-5p regulated loss of FBP1 altered cell function and fatty acid metabolism in Eca109 cells

**Figure 5:**
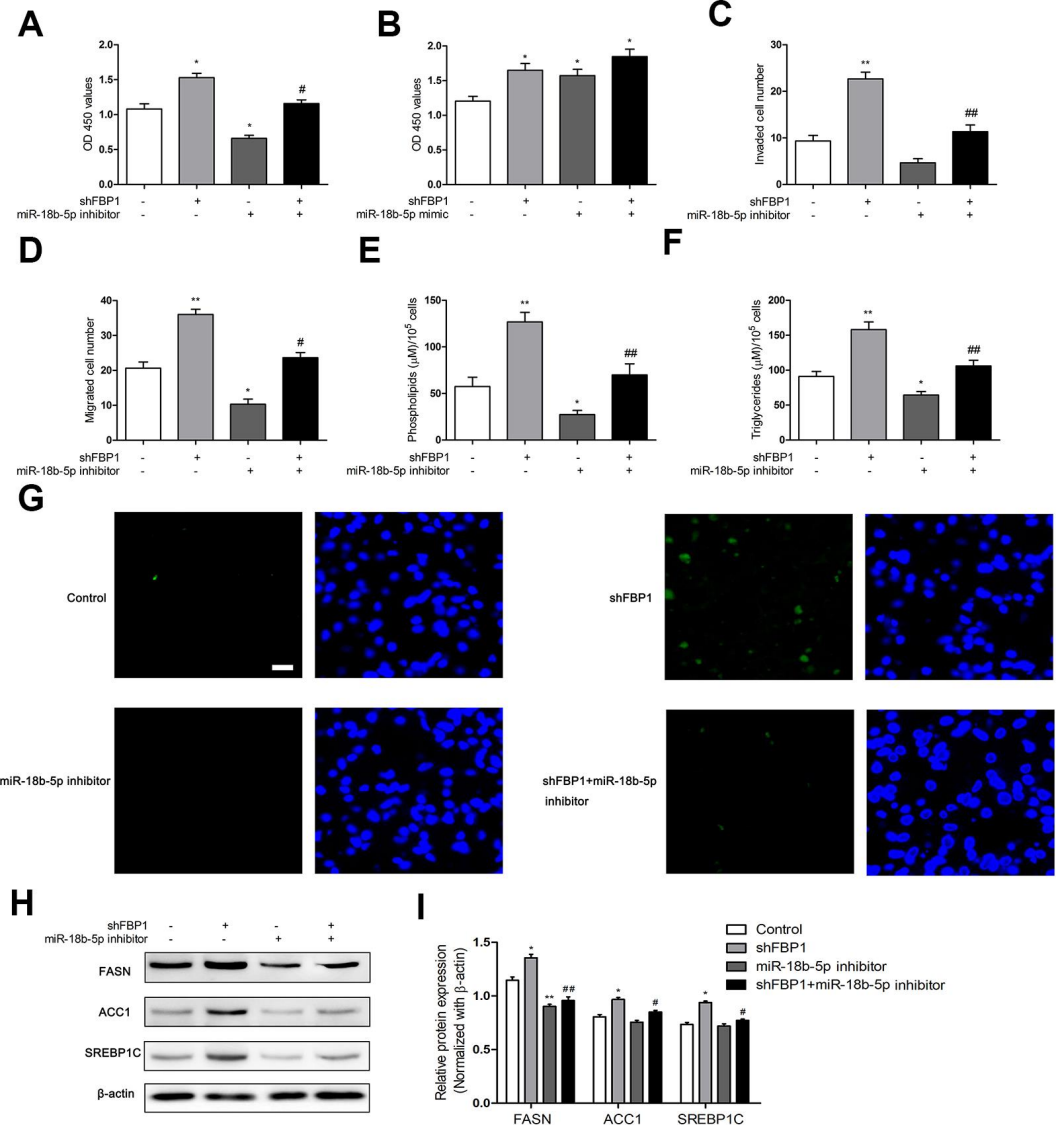
**MiR-18b-5p inhibitor regulated loss of FBP1 induced fatty acid metabolism in Eca109 cells.** (**A**) The Eca109 cell proliferation was determined following treatment with shFBP1 and/or miR-18b-5p inhibitor. (**B**) The Eca109 cell proliferation was determined following treatment with shFBP1 and/or miR-18b-5p mimic. (**C**) The Eca109 cell invasion was determined following treatment with shFBP1 and/or miR-18b-5p inhibitor. (**D**) The Eca109 cell migration was determined following treatment with shFBP1 and/or miR-18b-5p inhibitor. (**E**) The content of phospholipids was determined following treatment with shFBP1 and/or miR-18b-5p inhibitor. (**F**) The content of triglycerides was determined following treatment with shFBP1 and/or miR-18b-5p inhibitor. (**G**) The neutral lipids content was determined following treatment with shFBP1 and/or miR-18b-5p inhibitor by staining with BODIPY 493/503 dye (green) and DAPI (blue) in Eca109 cells, scale bar= 20μm. (**H**, **I**) The protein expression and quantification of FASN, ACC1 and SREBP1C was determined following treatment with shFBP1 and/or miR-18b-5p inhibitor. *p< 0.05 VS control, **p< 0.01 VS control, # p< 0.05 VS shFBP1, ## p< 0.01 VS shFBP1.

To explore the effect of miR-18b-5p regulated FBP1 on ESCC cell function, Eca109 cells were treated with miR-18b-5p inhibitor and/or shFBP1. The results showed that miR-18b-5p inhibitor combined with shFBP1 treatment effectively reversed the enhanced proliferation induced by shFBP1 transfection (Figure 5A). On the other hand, miR-18b-5p mimic combined with shFBP1 treatment did not significantly changed shFBP1 induced proliferation of Eca109 cells (Figure 5B). Moreover, the cell migration and invasion were significantly decreased by the combination treatment of miR-18b-5p inhibitor and shFBP1 when compared with shFBP1 alone treatment (Figure 5C, 5D). To further explore the effect of miR-18b-5p on FBP1 regulated fatty acid metabolism in ESCC cells, the contents of phospholipids, triglycerides and neutral lipids were determined following combination treatment of miR-18b-5p inhibitor and shFBP1. The results showed that miR-18b-5p inhibitor combined shFBP1 treatment lead to a significant decrease of the contents of phospholipids and triglycerides when compared with shFBP1 alone treatment (Figure 5E, 5F). Consistently, the content of neutral lipids was obviously decreased following combination treatment of miR-18b-5p inhibitor and shFBP1, which reverse the effect of shFBP1 on the content of neutral lipids (Figure 5G). In addition, miR-18b-5p inhibitor combined shFBP1 treatment also reduced the protein expression of FASN, ACC1 and SREBP1C, which enhanced by the treatment of shFBP1 (Figure 5H, 5I).

## DISCUSSION

Metabolic reprogramming has been defined as one of the hallmark tumor features [31, 32]. FBP1 plays an important role in the tumorigenesis and progression of several cancers [20, 23, 33]. FBP1 is also considered to be a potential marker of cancer and a prognostic marker [34, 35]. In addition, previous studies have shown that low levels of FBP1 are associated with low cancer survival rates and high relapse rates [36, 37]. However, the expression of FBP1 in ESCC and its tentative function are not clear. Here, in this study, we also found the decreased expression of FBP1 in ESCC patients. To further explore the mechanism of loss of FBP1 mediating ESCC cell function, FBP1 was down-regulated by transfection of shFBP1 in ESCC cells. We found loss of FBP1 promoted ESCC cell proliferation, migration and invasion *in vitro*. It is well known that FBP1 can reduce glucose uptake, increases oxygen consumption, and reduce lactic acid production. Silencing FBP1 can significantly promote the proliferation and metastasis of cancer cells [38, 39]. On the other hand, fatty acid metabolism also involves the cell proliferation and metastasis in various cancers [40, 41]. However, whether the decreased expression of FBP1 participated in the regulation of fatty acid metabolism remains unclear. Interestingly, we demonstrated that loss of FBP1 enhanced fatty acid metabolism in ESCC cells. The content of phospholipids, triglycerides and neutral lipids were significantly increased following down-regulation of FBP1. In addition, loss of FBP1 also enhanced the protein expression levels of fatty acid metabolism related FASN, ACC1 and SREBP1C. These results suggested that loss of FBP1 might promote ESCC proliferation, migration, and invasion by regulating fatty acid metabolism.

In recent years, there has been continuous evidence that miRNA is involved in the tumorigenesis and progression of ESCC [42, 43]. Previous study has shown that miR-373 promotes the development of ESCC by targeting LATS2 and OXR1 [44]. Harada K. et al. demonstrated that suppressor miR-145 expression was regulated by hypermethylation of the miR-145 promoter region in ESCC [45]. Moreover, miR-17, miR-18a, and miR-19a were showed to serve as potential unfavorable prognostic biomarkers in ESCC [46]. In this study, we also found miR-18B-5P was involved in FBP1 mediated cell function. Interestingly, FBP1 was directly targeted by miR-18B-5P in Eca109 cells. To explore the effect of miR-18b-5p regulated FBP1 on ESCC cell function and fatty acid metabolism, Eca109 cells were treated with miR-18b-5p inhibitor and shFBP1. We found the cell proliferation, migration and invasion were effectively reversed and the contents of phospholipids, triglycerides and neutral lipids were counteracted by the combined treatment of miR-18b-5p inhibitor and shFBP1, which induced by loss of FBP1. In addition, the fatty acid metabolism related protein expression of FASN, ACC1 and SREBP1C, which enhanced by the treatment of shFBP1, was also decreased following treatment of miR-18b-5p inhibitor and shFBP1. These results suggested that miR-18b-5p regulated cell function and fatty acid metabolism through targeting FBP1 in ESCC cells.

In conclusion, loss of FBP1 promoted ESCC cell proliferation, migration, and invasion *in vitro*, which was regulated by miR-18b-5p. Moreover, loss of FBP1 enhanced fatty acid content and fatty acid metabolism related proteins expression in ESCC cells. In addition, inhibition of miR-18b-5p counteracted the effect of loss of FBP1 on the cell function and fatty acid metabolism. These findings explored a detailed molecular mechanism of tumorigenesis and progression of ESCC and might provide a potential novel method to treat ESCC in clinic.

## MATERIALS AND METHODS

### Clinical samples

The clinical ESCC samples used in this study were histopathologically and clinically diagnosed at the Shanghai Chest Hospital affiliated to Shanghai Jiao Tong University with written consent and approval from the institutional research ethics committee. The ESCC tissues (21 cases) and adjacent non-ESCC tissues (21 cases) were collected for the subsequent experiments.

### Immunohistochemistry

Sections were dewaxed in xylene and rehydrated in a graded series of ethanol, followed by antigen retrieval sodium citrate buffer. Immunohistochemical staining was carried out by the primary antibody against FBP1 (Abcam), followed by treatment with the secondary detection antibody. Then the samples were washed and observed using an inverted microscope (Zeiss). The Image J (NIH) was used for the quantification of immunohistochemical staining.

### Human ESCC cell lines

Eca109 and ec9706 human ESCC cell lines were obtained from the cell bank center of our institute. The cell line authentication was performed and the STR analysis of two cells was consistent with the STR data from China Infrastructure of Cell Line Resources database. Cells were cultured in DMEM (Gibco, USA) with 10% fetal bovine serum and maintained at 37° C, 95% humidity, and 5% CO_2_.

### MiRNA, lentivirus construction, and transfection

The miR-18b-5p mimic, inhibitor, the controls and shFBP1 were obtained from Sangon Biotech (Shanghai, China). shFBP1 sequence was cloned into pLKO vector (Addgene, #10878). The sequence of shFBP1 was 5’-CGACCTGGTTATGAACAT GTT-3’. The cell transfection was performed using lipofectamine RNAiMAX (Invitrogen) or Lipofectamine 3000 Reagent (Invitrogen) according to the manufacturer’s instruction. ESCC cells were cultured in 6-well plate for 12h, and then received miR-18b-5p mimic, inhibitor, the controls or shFBP1 for indicated time point.

### Quantitative real-time PCR

Total RNA was isolated from ESCC cells using TRIzol reagent (Invitrogen). Next, the RNA was reversely transcribed to cDNA using a PrimeScript RT Master Mix Kit (Takara Bio). A SYBR Green PCR Master Mix (Invitrogen) was used to assess the FBP1 expression using a 7900 real-time PCR system (Applied Biosystems, Life Technologies). β-actin was used as an internal control. Fold changes for the expression levels of FBP1 were calculated using the comparative cycle threshold (CT) method (2^-ΔΔCT^). The results were performed in triplicate in real-time quantitative RT-PCR. The primers were as follows, FBP1 forward: 5’-CTCTATGGCATTGCTGG TTCT-3’; FBP1 reverse: 5’-TCCACTATGATGGCGTGTTTAT-3’.

### Western blot analysis

The protein lysates of ESCC cells were isolated after the cells were treated with shFBP1. The concentration of the protein was quantified and transferred the into nitrocellulose (NC) membranes. The NC membranes were blocked, and incubated with the primary antibody for 2h at room temperature. Finally, the protein expression was visualized using the chemiluminescence detection system. The primary antibodies against FASN (1:1000; R&D Systems), ACC1 (1:1000; Cell Signaling Technology), SREBP1C (1:2000; Abcam), FBP1 (1:1000; Abcam), a secondary antibody (anti-rabbit IgG, 1:7500; Cell Signaling Technology), and β-actin antibody (1:5000; Abcam) were used to determine the indicated protein expression. The Image J (NIH) was used for the quantification of protein expression.

### Cell proliferation assay

ESCC cell proliferation was examined using a CCK-8 assay kit (Dojindo, Japan). Briefly, cells were transfected with shFBP1 using Lipofectamine 3000 Reagent. After different time point of transfection, cells were added CCK-8 and incubated for 2h. The absorbance at 450 nm was measured with a microplate reader (ELX800, BioTeK, USA).

### Cell migration and invasion assays

The capability of cell migration and invasion were analyzed using non-Matrigel-coated (BD Falcon, BD Biosciences, USA) or Matrigel-coated transwell cell culture chambers (BD Matrigel Invasion Chamber, BD Biosciences, USA), 8-μm pore size. Briefly, cells were seeded on the upper chambers with serum-free medium. The lower chambers were added the medium contained 10% FBS. After culturing at 37° C and 5% CO2 for 48 h, cells on the upper side of the chamber were migrated and invaded into the lower chamber. The migration and invasion cells number was counted in three randomly selected areas under a light microscope.

### Immunoprecipitation assays

Cells were washed in ice-cold PBS, lysed in 500μl co-IP buffer, and incubated with the primary antibodies at 4° C for 12h. 40μl of 50% slurry of protein A-Sepharose was added to the sample. The mixtures were incubated at 4° C for 4h and then subject to brief centrifugation. The pellets were washed with PBS for three times and suspended in 0.5 ml Tri Reagent (Sigma). The elutes were subject to reverse transcription and PCR to detect the presence of the binding miR-18b-5p.

### RNA pull-down analysis

Cells were incubated with biotin (Bio)-labeled miR-18b-5p or Bio-labeled negative control (NC) (GenePharma, Shanghai) at 37° C for 4 h. M-280 Streptavidin Dynabeads (Life Technologies) were added per 100 pmol of biotin-DNA oligos, and the mixture was then rotated for 30 min at 37° C. Beads were captured by magnets (Life Technologies) and washed five times. Each experiment was carried out in triplicate.

### Dual-luciferase reporter assay

To investigate the relationship between FBP1 and miR-18b-5p, a luciferase reporter assay was performed. Cells were seeded and co-transfected with reporter of either wild type (wt) or mutant type (mut) and pRL-TK vector (Promega), an internal control using Lipofectamine 3000 (Invitrogen). Forty-eight hours after transfection, cells were collected for analysis using a dual-luciferase reporter assay kit (Promega).

### Immunofluorescence staining

The lipophilic fluorescence dye BODIPY 493/503 (Invitrogen) was employed for evaluate the neutral lipid accumulation. Briefly, cells were fixed with 4% paraformaldehyde and stained with BODIPY 493/503 for 1h at room temperature, and then nuclei were DAPI for 15 min. The results of immune staining were detected using a fluorescence microscope (Olympus, Tokyo, Japan).

### Flow cytometry

Cells were fixed with 4% paraformaldehyde for 20 min at room temperature. Cells were stained with 1 mL PBS containing BODIPY 493/503 for 15 min at room temperature, and washed before being analyzed on a flow cytometer (BD Biosciences) and FlowJo Software.

### Quantification of phospholipids and triglycerides

The content of phospholipids and triglycerides were determined in ESCC cells by EnzyChrom™ phospholipids assay kit (BioAssay Systems, Hayward, CA, USA) and EnzyChrom™ triglycerides assay kit (BioAssay Systems, Hayward, CA, USA) respectively.

### Statistical analysis

SPSS 17.0 software (Chicago, IL, USA) was used for statistical analysis in this study. Data were presented as means ± SD, which were collected from at least 3 independent experiments. Two-tailed Student’s t-test was used for comparisons of two independent groups. P < 0.05 was considered statistically significant.
